# Association of vaccine status, reinfections, and risk factors with Long COVID syndrome

**DOI:** 10.1038/s41598-024-52925-4

**Published:** 2024-02-02

**Authors:** Maria Elena Romero-Ibarguengoitia, Juan Francisco Rodríguez-Torres, Arnulfo Garza-Silva, Andrea Rivera-Cavazos, Devany Paola Morales-Rodriguez, Mauricio Hurtado-Cabrera, Ricardo Kalife-Assad, Diana Villarreal-Parra, Alejandro Loose-Esparza, Juan José Gutiérrez-Arias, Yaressi Guadalupe Mata-Porras, Daniela Abigail Ojeda-Salazar, Miguel Angel Sanz-Sánchez, Arnulfo González-Cantú, Elena Azzolini, Maria Rescigno

**Affiliations:** 1 Research Department, Hospital Clínica Nova de Monterrey, San Nicolás de los Garza, Nuevo León México; 2 Escuela de Medicina, Vicerrectoría de Ciencias de la Salud, Universidad de Monterrey, San Pedro Garza García, Nuevo León México; 3 Internal Medicine Department, Hospital Clínica Nova de Monterrey, San Nicolás de los Garza, Nuevo León México; 4 IRCCS Humanitas Research Hospital, Via Manzoni 56, 20089 Rozzano, Milan Italy; 5 Department of Biomedical Sciences, Humanitas University, Via Rita Levi Montalcini 4, 20072 Pieve Emanuele, Milan Italy

**Keywords:** Vaccines, Virology

## Abstract

The COVID-19 pandemic had a profound global impact, characterized by a high fatality rate and the emergence of enduring consequences known as Long COVID. Our study sought to determine the prevalence of Long COVID syndrome within a population of Northeastern Mexico, correlating it with patients' comorbidities, number of COVID-19 reinfection, and vaccination status. Employing an observational cross-sectional approach, we administered a comprehensive questionnaire covering medical history, demographics, vaccination status, COVID-related symptoms, and treatment. Our participant cohort included 807 patients, with an average age of 41.5 (SD 13.6) years, and women accounting 59.3% of the cohort. The follow-up was 488 (IQR 456) days. One hundred sixty-eight subjects (20.9%) met Long COVID criteria. Long COVID-19 was more prevalent when subjects had reinfections (p = 0.02) and less frequent when they had a complete vaccination scheme (p = 0.05). Through logistic regression, we found that male gender (OR 0.5, p ≤ 0.001), blood types of AB− (OR 0.48, p = 0.003) and O− (OR 0.27, p ≤ 0.001) in comparison with A+ and two doses of vaccines (OR 0.5, p = 006) to be protective factors against Long COVID; while higher BMI (OR 1.04, p = 0.005) was a risk factor. We saw that the prevalence of Long COVID was different within vaccinated patients and specific blood types, while being female and a higher BMI were associated with an increased risk of having long-COVID.

## Introduction

The COVID-19 global pandemic caused by the *severe acute respiratory syndrome Coronavirus-2* started in 2019, and has been responsible for more than 700 million cases and nearly 7 million deaths^1^. The established pathogenetic mechanism by which the virus infiltrates its host involves the binding of its spike protein to angiotensin-converting enzyme 2 (ACE2). This, in turn, triggers an inflammatory cascade orchestrated by both cellular and humoral immune responses, culminating in a spectrum of acute symptoms that can range from a mild flu-like illness to severe acute respiratory distress syndrome (ARDS) accompanied by multiorgan failure^2^. Recent studies have shown that subjects that recovered from acute infection could develop a reinfection^3^. Since the introduction of vaccines against SARS-CoV-2, the incidence and severity of the disease have decreased globally. The main vaccines used in the Mexican population were Pfizer-BioNTech BNT162b2, Moderna Spikevax, Oxford/AstraZeneca ChAdOx1-S, Janssen Ad26.COV2.S, Sinovac-CoronaVac, CanSino Biologics Ad5-nCoV-S, and Sputnik V. The accessibility was different depending on the region and age. Their efficacy for prevention of acute or severe diseases varies from > 90% (mainly those of messenger RNA) to as low as 50%^4^. Nowadays, the community knows the possible vaccine combinations that enhance their response against the acute infection^5^.

Long COVID syndrome, or post-acute COVID-19 syndrome, is defined, according to the Center for disease control and prevention (CDC), as a clinical condition in which the symptoms associated with acute infection of COVID-19 persist for a duration of 4 weeks or more following the initial diagnosis. Several signs and symptoms are associated with this disease, affecting different organic systems, and they vary from persistent coughing to cardiovascular complications^6–8^. This syndrome has been associated with multiple factors, mainly from the host and the severity of the acute disease. The main risk factors associated are female sex, hypertension, obesity, and immunocompromised patients^7,8^. The pathophysiological mechanisms are not well established yet, but it is believed that it is linked to chronic inflammation caused by the persistence of viral load, lymphopenia, and autoimmune factors, which result in long-term tissue damage. The theory by which these autoimmune factors are present is believed to be associated to persistence of spike proteins in the blood stream^9^. Additionally, there has been an observed association with the presence of inflammatory cytokines: IL-6, tumor necrosis factor alpha, nitric oxide, and calcium channel modulating activity^10^.

Numerous cohort and observational studies have confirmed a relationship between vaccination status within a group experiencing Long COVID and a control group without symptoms; most of them evaluated the relation with BNT16b2 vaccine or ChAdOx1-S. One such investigation, involving 28,356 adults aged 18–69 in England, examined those who had tested positive for COVID-19 and subsequently received vaccination. Within this cohort, 23.7% reported symptoms indicative of Long COVID syndrome. A parallel study in France followed up with 155 adults who had experienced COVID-19 symptoms. Over a 120-day span, 16.6% of the vaccinated individuals experienced a complete resolution of symptoms, in contrast to 7.5% of those who remained unvaccinated^11,12^. An additional study, also conducted in the United Kingdom, explored the connection between post-vaccine antibody titers and post-COVID syndrome. Within this context, 61% reported no alteration in symptoms, 21% noted worsening, and 16% experienced improvement 2 weeks following vaccination^13^. A systematic review by the UK Health Security Agency, encompassing 16 observational studies conducted in 2022, concluded that vaccines were effective in preventing Long COVID, particularly with earlier virus variants, largely by averting acute infections^14^. In a meta-analysis encompassing 12 global studies and a collective patient pool of 614,392 individuals, variations in the reduction of odds ratios (OR) were observed across different vaccination doses. However, the significance of this information was deemed inconclusive due to substantial heterogeneity among the studies^15^. In Italy, a different study determined a Long COVID syndrome prevalence rate of 31%, which exhibited a decline across the initial three pandemic waves and in conjunction with the BNT162b2 vaccine^16^. There is scare information that has evaluated different vaccination schemes with/without booster and the prevalence of Long COVID.

In Mexico, a case–control study was undertaken involving 219 patients across three different cities. This study aimed to compare the risk of developing persistent COVID-19 symptoms between individuals who had contracted symptomatic infection and those who remained symptom-free. The findings revealed an elevated risk of Long COVID when the infection was symptomatic, with a relative risk (RR) of 2.43 (90% CI 1.78, 3.33). Among the symptoms reported, those associated with a heightened risk of persisting after a COVID-19 infection included chills, dyspnea, new anosmia or dysgeusia, nausea or vomiting, cough, and red eyes^17^. In another study focusing on symptom clusters within a central Mexican hospital, a prevalence of COVID-19 symptoms was observed at 23.8% within 30 days following hospital discharge. After 90 days of follow-up, many symptoms displayed a tendency to decrease, except for specific symptoms such as alopecia, rhinorrhea, lacrimation, lack of concentration, paresthesia, arthralgias, and memory loss^18^. The comprehensive investigation conceded no existing findings about the relationship between vaccines in Mexico and the prevalence of Long COVID. Given the considerable diversity in vaccine administration within the Mexican population, it becomes crucial to explore whether these findings diverge from studies conducted in regions where vaccine distribution was more selective. Also, there is scare information of the relation with Long COVID, reinfection in Mexico.

This study aimed to determine the prevalence of Long COVID syndrome in a population living in Northeastern Mexico. Additionally, it aimed to explore potential differences in the occurrence of Long COVID based on vaccination status and the frequency of COVID-19 reinfections. Lastly, the study aimed to uncover any potential associations between clinical risk factors and the emergence of Long COVID within our specific region.

## Results

There was a total of 818 surveys applied; however, a total of 804 were analyzed after duplicates were removed and exclusion criteria applied. The median (IQR) follow-up of the patients from the first COVID-19 infection until survey application was 488.5 (456). They were a total of 477 (59.3%) women and 327 (40.6%) men with a mean (SD) age of 41.5 (13.6) years. There were 535 (66.5%) patients that had no history of any medical condition.

The most prevalent diseases were the presence of hypertension with 127 (15.8%) patients, type 2 diabetes with 122 (15.2%), and autoimmune disease with 54 (6.7%), which had not significant differences between subjects that developed Long COVID vs. the control group. Subjects that developed Long COVID had a statistically significant higher frequency of a history of allergies [n = 37 (31.4%) vs n = 81 (13.1%)], p = 0.022. The rest of the variables regarding the medical history by presence of Long COVID are addressed in Table 1.

**Table 1:** Patients medical history.

Variable (frequency)	Total n = 804 (%)	Without long COVID n = 618 (%)	With long COVID n = 186 (%)	p value
Patients without comorbidities	535 (66.5)	422 (68.3)	113 (21.1)	0.056
Hypertension	127 (15.8)	93 (15.0)	34 (18.3)	0.289
Type 2 diabetes	122 (15.2)	83 (13.9)	36 (19.4)	0.07
Allergies	118 (14.7)	81 (13.1)	37 (31.4)	0.022
Autoimmune disease^a^	54 (6.7)	38 (6.1)	16 (8.6)	0.241
Dyslipidemia^b^	21 (2.6)	14 (2.3)	7 (33.3)	0.261
Asthma	17 (2.10)	14 (2.3)	3 (1.6)	0.588
Rheumatologic diseasec	9 (1.1)	8 (1.3)	1 (0.5)	0.39
Hepatic steatosis	7 (0.9)	5 (0.8)	2 (1.1)	0.732
Type 1 diabetes	3 (0.4)	3 (0.5)	0 (0)	0.341
Heart failured	3 (0.4)	2 (0.3)	1 (0.5)	0.675
Immunosuppression	3 (0.4)	5 (0.5)	0 (0.0)	0.341

^a^Patient’s medical history between a group with and without Long COVID.Systemic lupus erythematosus, psoriasis, Sjörgen syndrome, hypothyroidism.

^b^Imbalance of serum levels of lipids: high levels of total cholesterol (> 200 mg/dL), high levels of low-density lipoprotein cholesterol (LDL-C) (> 100 mg/dL), high levels of triglycerides (> 150 mg/dL), and low levels of high-density lipoprotein (HDL) (Men < 40 mg/dL; women < 50 mg/dL).

^c^Rheumatoid arthritis, osteoarthritis, gouty arthritis.

dHistory of myocardial infarction, unstable angina, or coronary reperfusion.

Among the different vaccination schemes the surveyed had, the most common were the complete scheme with the Pfizer-BioNTech BNT162b2 plus a booster with 273 (34%) patients, of which the type of booster vaccine varied. After that, the second most common scheme was the Sinovac-CoronaVac regime plus booster with a total of 123 (14.1%) patients, followed the Oxford/AstraZeneca ChAdOx1-S complete regime plus booster with 110 (13.7%) patients. The least common vaccines that people received were the CanSino Biologics Ad5-nCoV-S (n = 22, 2.7%), Janssen Ad26.COV2-S (n = 8, 1.0%) and Sputnik V Gam-COVID-Vac regime had the least amount (n = 4, 0.5%). The rest of the variables regarding the most common vaccination schemes are addressed in Table 2.

**Table 2:** Most common vaccination groups in the population.

Vaccine group (n = 804)	Frequency (%)
Pfizer-BioNTech BNT162b2 regime plus booster*	273 (34.0)
Sinovac-CoronaVac regime plus booster*	113 (14.1)
Oxford/AstraZeneca ChAdOx1-S regime plus booster*	110 (13.7)
Pfizer-BioNTech BNT162b2 complete regime	70 (8.7)
Oxford/AstraZeneca ChAdOx1-S complete regime	48 (6.0)
Moderna/Spikevax mRNA-1273 regime plus booster*	47 (5.8)
Other schemes without Booster^a^	76 (9.4)
Other schemes plus Booster^b^	58 (7.2)

Vaccination schemes for Covid-19. *Booster regimes involve 1 or 2 booster shots added to the complete regime given by every vaccine distributor. In our population the boost shot consisted of BNT162b2, ChAdOx1-S, Moderna/Spikevax mRNA-1273, and CoronaVac.

^a^The “Other schemes without Booster” group is comprised of patients with groups of single vaccine dose of Pfizer-BioNTech BNT162b2 and Oxford/AstraZeneca ChAdOx1-S, or a complete scheme of Moderna/Spikevax mRNA-1273, Sinovac-CoronaVac, CanSino Ad5-nCOV; or a heterologous combination of the before mentioned different vaccines.

^b^The “Other schemes plus Booster” group involves complete vaccination scheme from CanSino Ad5-nCOV, Janssen Ad26.COV2-S or Sputnik V Gam-COVID-Vac or a heterologous combinations of Pfizer-BioNTech BNT162b2, Oxford/AstraZeneca ChAdOx1-S, Moderna/ Spikevax mRNA-1273, as the first or second dose, plus a booster of the before mentioned vaccines.

Of the surveyed patients, a total of 804 individuals experienced acute COVID-19 at least once since March 2020. Most of these patients were treated ambulatory, reflecting a mild disease (n = 674, 83%). Within this group, 168 (20.9%) patients met the criteria for Long COVID during their initial COVID-19 infection. The most prevalent symptom associated with Long COVID in this cohort was fatigue/tiredness, which was present in 50 (29.8%) patients. Other prevalent symptoms included alopecia in 35 (20.8%) patients, dysgeusia in 30 (17.9%) patients, and cough in 29 (17.3%) patients.

A significant difference was observed in the prevalence of Long COVID syndrome among distinct vaccination categories. Among the unvaccinated group, 73 (25.1%) individuals experienced Long COVID, the group with incomplete vaccination regimen included 16 (18.4%) individuals experienced Long COVID. Notably, the prevalence of Long COVID was lowest in the group with a complete vaccination regimen, with 43 (16%) individuals. The group that received a booster shot consisted of 36 (22.8%) individuals with Long COVID. This difference of overall Long COVID prevalence between vaccination status groups reached statistical significance, p = 0.05. Among patients experiencing anosmia as a symptom of Long COVID syndrome, a statistically significant difference in vaccination status was observed on patients with complete vaccination regime, p = 0.049. There was no other statistical difference between the vaccination groups and the isolated symptoms. The rest of the variables regarding diagnosis and symptoms of Long COVID within the first COVID-19 infection, along with the corresponding comparison to vaccination are addressed in Table 3.

**Table 3:** Long COVID Symptoms after first infection.

Long COVID symptoms	Prevalence n = 804 (%)	Without vaccination n = 291 (%)	Incomplete vaccination regime^a^ n = 87 (%)	Complete vaccination regime n = 268 (%)	Complete vaccination regime + Booster shot^b^ n = 158 (%)	*p *value^d^
Presence of Long COVID	168 (20.9)	73 (25.1)	16 (18.4)	43 (16)	36 (22.8)	0.050
Fatigue/tiredness	50 (29.8)	22 (7.5)	9 (10.3)	11 (4.1)	8 (5.1)	0.114
Other symptoms^c^	15 (25.6)	8 (25.8)	0 (0.0)	5 (9.6)	2 (6.67)	0.092
Alopecia	35 (20.8)	11 (61.1)	5 (83.3)	10 (76.9)	9 (89.1)	0.643
Dysgeusia	30 (17.9)	19 (19.8)	0 (0.0)	9 (18.8)	2 (9.1)	0.090
Persistent cough	29 (17.3)	9 (8.3)	5 (15.2)	13 (13.2)	12 (17.6)	0.289
Anosmia	23 (13.7)	15 (13.5)	0 (0.0)	8 (16.3)	0 (0.0)	0.049
Memory loss	18 (10.7)	7 (43.8)	1 (50)	5 (45.5)	5 (38.4)	1.000
Dyspnea	16 (9.5)	10 (16.7)	3 (21.4)	1 (4.5)	2 (18.2)	0.426
Insomnia	15 (8.9)	9 (36.0)	1 (20.0)	1 (11.1)	4 (33.3)	0.600
Migraine	12 (7.1)	6 (3.9)	0 (0.0)	3 (2.1)	3 (3.12)	0.616
Anxiety/depression	12 (7.1)	5 (25.0)	1 (33.3)	2 (25.0)	4 (44.4)	0.761
Muscle pain	9 (5.4)	5 (3.6)	1 (2.6)	2 (1.8)	1 (1.3)	0.809
Paresthesia	8 (4.8)	3 (50.0)	1 (50.0)	1 (50.0)	3 (60.0)	1.000
Hiporexia	8 (4.8)	4 (10.3)	0 (0.0)	2 (10.5)	2 (13.3)	1.000
Weight loss	8 (4.8)	3 (18.75)	2 (50.0)	1 (25.0)	2 (66.7)	0.292
Vertigo	6 (3.6)	3 (42.9)	0 (0.0)	3 (42.9)	0 (0.0)	0.197
Chest pain	6 (3.6)	5 (17.8)	0 (0.0)	0 (0.0)	1 (11.1)	0.524
Joint pain and/or swelling	6 (3.6)	4 (18.2)	0 (0.0)	1 (7.7)	1 (11.1)	0.581
Palpitations	4 (2.4)	2 (20.0)	0 (0.0)	1 (16.7)	1 (1.11)	1.000
Abdominal pain	3 (1.8)	1 (10.0)	0 (0.0)	0 (0.0)	2 (25.0)	0.619
Constipation	3 (1.8)	1 (8.3)	0 (0.0)	1 (11.1)	1 (25.0)	0.765
Pain during breathing	2 (1.2)	1 (8.3)	0 (0.0)	1 (25.0)	0 (0.0)	0.682
Nausea or vomiting	1 (0.6)	0 (0.0)	0 (0.0)	0 (0.0)	1 (14.3)	0.514
Diarrhea	1 (0.6)	1 (4.7)	0 (0.0)	0 (0.0)	0 (0.0)	1.000
Communication issues	1 (0.6)	0 (0.0)	0 (0.0)	0 (0.0)	1 (100.0)	1.000
Tremors	1 (0.6)	1 (20.0)	0 (0.0)	0 (0.0)	0 (0.0)	1.000
Cutaneous rash	1 (0.6)	0 (0.0)	0 (0.0)	1 (50.0)	0 (0.0)	1.000
Chewing/swallowing issues	1 (0.6)	1 (33.3)	0 (0.0)	0 (0.0)	0 (0.0)	0.299

Long COVID symptoms after first COVID-19 infection compared with their vaccination status.

^a^Incomplete vaccination regime applies only to those vaccine schemes that require at least two doses including Pfizer-BioNTech BNT162b2, Moderna/Spikevax mRNA-1273, Oxford/AstraZeneca ChAdOx1-S, Sinovac-CoronaVac, and Sputnik V Gam-COVID-Vac.

^b^In the booster shot, we included patients with 1 or 2 booster shots after the complete vaccination.

^c^Other symptoms included sore throat, rhinorrhea, glossodynia, dysphonia and cacosmia.

^d^Significance with Exact Fisher Test with Monte Carlo approximation.

A total of 188 patients experienced a second COVID-19 infection, of which 163 (86%) received ambulatory treatment and 28 (14.8%) individuals met the criteria for Long COVID syndrome. The prevailing symptoms within this group were fatigue/tiredness, affecting 8 (28.5%) patients, followed by persistent cough, which impacted 7 (25%) patients. Among the individuals who contracted the virus for a third time (24 in total, 91% received ambulatory treatment), only 4 (16.7%) of them fulfilled the criteria for Long COVID. The symptoms observed in this subset included fatigue/tiredness in 1 case (25%), migraine in 1 case (25%), alopecia in 1 case (25%), and anosmia in 1 case (25%). Notably, no significant distinction was observed between the vaccination status and the presence of Long COVID in either of these groups. Some of the same individuals that had Long COVID during the first infection also developed it after the second infection and during the third infection. But from the 28 cases in the second infection 17 were new cases and no new Long COVID cases were found during the third infection. The rest of the variables regarding diagnosis and symptoms of Long COVID within the second and third COVID-19 infection, along with the corresponding comparison to vaccination are addressed in Tables 4 and 5. When comparing the number of reinfection and the presence of Long COVID through chi-square text there was a higher prevalence in the group with more reinfections, p = 0.02.

**Table 4:** Long COVID symptoms after second infection.

Long COVID symptoms	Prevalence n = 188 (%)	Without vaccination n = 20 (%)	Incomplete vaccination regime^a^ n = 29 (%)	Complete vaccination regime n = 87 (%)	Complete vaccination regime + Booster shot^b^ n = 52 (%)	*p value* ^d^
Presence of Long COVID	28 (14.9)	6 (30.0)	2 (6.9)	12 (13.8)	8 (15.4)	0.183
Fatigue/tiredness	8 (28.6)	2 (10.0)	1 (3.4)	4 (4.5)	1 (1.9)	0.444
Persistent cough	7 (25.0)	0 (0.0)	1 (8.3)	6 (18.2)	0 (0.0)	0.131
Dysgeusia	4 (14.3)	0 (0.0)	0 (0.0)	2 (25.0)	2 (50.0)	0.405
Muscle pain	3 (10.7)	1 (12.5)	0 (0.0)	1 (3.3)	1 (4.3)	0.568
Anxiety/depression	3 (10.7)	0 (0.0)	0 (0.0)	0 (0.0)	3 (100.0)	0.100
Anosmia	3 (10.7)	2 (50.0)	0 (0.0)	0 (0.0)	0 (0.0)	0.498
Memory loss	2 (7.1)	0 (0.0)	0 (0.0)	1 (33.3)	1 (100.0)	0.189
Alopecia	2 (7.1)	0 (0.0)	0 (0.0)	1 (50.0)	1 (33.3)	1.000
Dyspnea	2 (7.1)	1 (33.3)	0 (0.0)	0 (0.0)	1 (50.0)	0.678
Palpitations	2 (7.1)	2 (100.0)	0 (0.0)	0 (0.0)	0 (0.0)	0.333
Migraine	1 (7.1)	0 (0.0)	0 (0.0)	1 (2.6)	0 (0.0)	1.000
Insomnia	1 (7.1)	0 (0.0)	0 (0.0)	0 (0.0)	1 (50.0)	0.506
Chest pain	1 (7.1)	0 (0.0)	0 (0.0)	0 (0.0)	1 (33.3)	1.000
Hiporexia	1 (7.1)	0 (0.0)	0 (0.0)	0 (0.0)	1 (33.3)	1.000
Nausea or vomiting	1 (7.1)	0 (0.0)	0 (0.0)	0 (0.0)	1 (100.0)	0.283
Constipation	1 (7.1)	0 (0.0)	0 (0.0)	0 (0.0)	1 (50.0)	0.506
Weight loss	1 (7.1)	0 (0.0)	0 (0.0)	0 (0.0)	1 (100.0)	1.000
Other symptoms ^c^	1 (7.1)	0 (0.0)	0 (0.0)	1 (8.3)	0 (0.0)	0.580

Long COVID symptoms after second COVID-19 infection compared with their vaccination status.

^a^Incomplete vaccination regime applies only to those vaccine schemes that require at least two doses including Pfizer-BioNTech BNT162b2, Moderna/Spikevax mRNA-1273, Oxford/AstraZeneca ChAdOx1-S, Sinovac-CoronaVac, and Sputnik V Gam-COVID-Vac.

^b^In the booster shot, we included patients with 1 or 2 booster shots after the complete vaccination.

^c^Other symptoms included sore throat, rhinorrhea, glossodynia, tinnitus, metrorrhagia, dysphonia and cacosmia.

^d^ Significance with Exact Fisher Test with Monte Carlo approximation.

**Table 5:** Long COVID symptoms after third reinfection.

Long COVID symptoms	Prevalence n = 24 (%)	Without vaccination n = 1 (%)	Incomplete vaccination regime^a^ n = 2 (%)	Complete vaccination regime n = 8 (%)	Complete vaccination regime + Booster shot^b^ n = 13 (%)	*p value* ^c^
Presence of Long COVID	4 (16.7)	0 (0.0)	0 (0.0)	1 (12.5)	3 (23.1)	1.000
Fatigue/tiredness	1 (4.2)	0 (0.0)	0 (0.0)	1 (12.5)	0 (0.0)	0.459
Migraine	1 (9.1)	0 (0.0)	0 (0.0)	0 (0.0)	1 (20.0)	1.000
Alopecia	1 (100.0)	0 (0.0)	0 (0.0)	0 (0.0)	1 (100.0)	-

Long COVID symptoms after third COVID-19 infection compared with their vaccination status.

^a^Incomplete vaccination regime applies only to those vaccine schemes that require at least two doses including Pfizer-BioNTech BNT162b2, Moderna/Spikevax mRNA-1273, Oxford/AstraZeneca ChAdOx1-S, Sinovac-CoronaVac, and Sputnik V Gam-COVID-Vac.

^b^In the booster shot, we included patients with 1 or 2 booster shots after the complete vaccination.

^c^Significance with Exact Fisher Test with Monte Carlo approximation.

Additionally, a logistic regression model was executed to pinpoint predictors of the Long COVID syndrome. The dependent variable was the presence of Long COVID, and the independent variables were age, gender, Body Mass index (BMI), blood type, number of reinfections and the number of vaccine schemes in the first infection. The findings showed a reduction in the risk to present Long COVID in male gender (OR 0.520, p = 0.001), AB− (OR 0.481, p = 0.003), and blood type O− (OR 0.276, p ≤ 0.001) in comparison with A+ and the presence of two vaccines (OR 0.551, p = 0.006); while a higher BMI (OR 1.043, p = 0.005) was the main risk factors. Reinfection had a marginal effect (p = 0.097) and there was no effect on age or the presence of allergies. Further details regarding the remaining variables in this model can be found in Table 6.

**Table 6:** Logistic regression model for risk factor to develop Long COVID.

n = 802	β	Standard error	OR	*p *value	CI 95%	
Age	− 0.002	0.007	0.998	0.815	0.985	1.012
Male sex	− 0.653	0.189	0.520	< 0.001	0.359	0.754
BMI	0.042	0.015	1.043	0.005	1.013	1.074
Allergies	0.330	0.235	1.398	0.145	0.89	2.202
A+ (n = 111)						
A− (n = 9)	− 1.385	1.117	0.250	0.215	0.028	2.238
B+ (n = 34)	− 0.785	0.464	0.456	0.091	0.184	1.151
B− (n = 5)	− 0.875	1.158	0.417	0.45	0.043	4.35
AB+ (n = 13)	− 1.354	0.8	0.258	0.092	0.053	1.250
AB− (n = 321)	− 0.731	0.244	0.481	0.003	0.298	0.77
O+ (n = 21)	0.011	0.507	0.989	0.982	0.366	2.671
O− (n = 288)	− 1.289	0.263	1.799	< 0.001	0.165	0.462
Number of reinfections	0.587	0.354	0.22	0.097	0.899	3.6
Without vaccine (n = 290)						
One vaccine dose (n = 87)	− 0.263	0.300	0.769	0.382	0.427	1.385
Two vaccine doses (n = 268)	− 0.596	0.219	0.551	0.006	0.359	0.846
Three vaccine doses (n = 157)	− 0.369	0.244	0.691	0.130	0.429	1.115
Intercept	1.736	0.688	0.176	0.012		

Nagelkerke R^2^ = 0.117.

## Discussion

This cross-sectional observational study showed a prevalence of Long COVID of 20.9% during the first infection, 14.8% during second reinfection and 16.7% after third reinfection. There was a reduced prevalence of Long COVID when patients had a complete vaccination scheme that could include one or two boost of any of the studied vaccines. Additional risk factors for Long COVID were being female, BMI, and the number of reinfections. The protective factors were blood type AB− and O− in comparison with A+.

The overall prevalence of Long COVID of 20.9%, aligns with the range of prevalence documented in studies from various countries^6,12,15,16,18^. The most prevalent symptoms overall were fatigue, hair loss, cough, sore throat, rhinorrhea, dyspnea, insomnia, and migraine. These results are consistent compared with other studies, although some place memory loss and concentration issues among the common symptoms associated with Long COVID and we did not find these issues^8^.

In relation to patient comorbidities associated with the presence of Long COVID, our investigation revealed a higher prevalence of history of allergies in the Long COVID group; however, in the regression model it did not show to be a risk factor. Nonetheless, when contrasting the outcomes between the non-Long COVID group and the Long COVID group, similar proportions were found including Hypertension, Type 2 Diabetes, Autoimmune Diseases, and Dyslipidemia. Additionally, through our regression model we found other risk factors such as being female, overweight or obese and A+ blood type in comparison with O− or AB-. In the literature there are heterogeneity of results, some studies confirm our findings, while other studies do not, this could be in relation to the different design of the studies and in relation that studies are conducted in different populations and that Long COVID distributes differently according to several sociodemographic status^9,19^

Concerning the vaccine's effectiveness in averting Long COVID symptoms, statistically significant findings emerged for patients who received a two-dose scheme before COVID-19 infection showing a lowered prevalence of Long COVID; also patients with a booster dose had a lower prevalence of anosmia. However, no distinction was observed between the vaccinated and unvaccinated groups during second and third reinfections. Nevertheless, an important decrease in the prevalence of Long COVID syndrome was evident among patients with multiple infections, compared to the prevalence of the first infection. This phenomenon might be linked to a favorable correlation with their vaccination status, the existence of less aggressive viral variants, or the plausible influence of acquired natural immunity from prior infections. The Cohort studied by Richard et al., showed that vaccination after COVID-19 infection was associated to a reduction in symptoms of Long COVID at 6 months^20^. Furthermore, in a longitudinal study conducted by Azzolini et al., the prevalence of Long COVID showed a decline within the BNT162b2 vaccinated group when contrasted with the unvaccinated cohort^16^. The limitation of these studies is that the population was vaccinated with ChAdOx1nCoV-19 Oxford-AstraZeneca, Ad26.COV2.S Janssen COVID-19, BNT162b2 Pfizer-BioNTech and mRNA-1273 Moderna. Our study adds an analysis of a population with more diverse scheme of vaccination showing that all combinations are related to a lower prevalence of Long COVID in the first infection.

In regards of the COVID-19 reinfection, we saw a notable decrease in the prevalence of Long COVID compared to the total of patients that tested positive. During the first infection the prevalence was 20.9%, in the second infection was 14.9%, and in the third 16.7%. Our Chi-square model showed a higher proportion of Long COVID in subjects with multiple reinfections; however, our regression modeled showed marginal effects. In the literature there are contradictory results. The study of Bowe et al. reported that after two or more reinfection and a follow-up of 6 months, there was an increase in pulmonary, cardiovascular, hematological, musculoskeletal, gastrointestinal, kidney and neurological sequalae regardless of vaccination status. The limitation of the study was that the subjects were predominantly Caucasian males^21^. A study conducted in the Amazonas that included 1371 patients that were infected with SARS-COV-2 showed that reinfection was associated with higher number of symptoms of Long COVID^22^. In a separate prospective study carried out in Italy by Peghin et al., an observation spanning 2 years was conducted on patients who had experienced a COVID-19 infection, there was discernible influence of reinfection on the dynamics of Long COVID syndrome^23^.

Within our predictive model, the male sex, as well as blood types AB− and O−, and two vaccination schemes before first COVID-19 infection emerged as protective factors against the development of Long COVID syndrome. Conversely, female sex and higher BMI were associated with an elevated likelihood of experiencing Long COVID following a COVID-19 infection. Finally, we did not find any correlation with age. In the study conducted in Amazonas, BMI and female gender were also related to be risk factors^22^. Female risk was also confirmed by Bechmann et al.^24^ and other researchers^9,18^. In relation to blood type. Other studies have reported that A+ is a risk factor for Long COVID; we found this association but only when compared with AB− and O− blood types. Caution must be taken when interpreting the significance of blood types A− and B− in relation to Long COVID in our model, as the limited number of patients with these blood types in our subjects makes it challenging to draw definitive conclusions about their potential risk or protective effects against the condition. Finally, in a review conducted by Abul et al., it was observed that the prevalence of Long COVID rises among older adults. Approximately 1 in 4 older adults may experience this syndrome, contrasting with 1 in 5 among the younger population^25^; in our study we could not confirm this finding.

The value of this research study is that it analyzes the risk factors for Long COVID in a Northern population of Mexico that is exposed to different brands and types of vaccines that are not frequently reported in the literature. These subjects could suffer of multiple reinfections confirmed by SARS-COV 2 PCR and analyzes other risk factors such as gender, age, BMI, blood type and comorbidities.

The primary limitation encountered during this investigation revolves around the absence of a population with moderate to severe disease; nearly all subjects included in the study exhibited mild symptoms. Research indicates that individuals with more severe illnesses, necessitating invasive treatments like ECMO, often grapple with emotional and physical consequences in contrast to those who experience milder disease and treatment^18,26^.

Another limitation stemmed from the lack of information pertaining to the specific viral variant responsible for the acute infection. This data gap hinders our ability to explore potential associations with the development of Long COVID syndrome. This shortfall is primarily attributed to the limited molecular testing that most patients underwent. Filling this gap could have provided valuable insights and facilitated additional correlations with disease prevalence. While it's possible to infer the variant based on the infection date, considering the prevailing prevalence during the specific wave of infection, this method remains inherently imprecise. An additional challenge encountered in this study pertained to the analysis of Long COVID symptoms following the second and third COVID-19 infections. This challenge stemmed from the limited number of subjects within each group, which resulted in potentially unreliable p values from the chi-square analysis.

A significant bias observed in our study relates to memory recall, as patients often struggled to recollect precise details. To address this, patients consulted family members and documents saved in their personal items to aid their recollection. We mitigated this issue by conducting individualized interviews through a questionnaire and requesting patients to present their vaccination certificates as validation.

In conclusion, our findings reveal a reduction in the prevalence of Long COVID infection among the vaccinated groups when compared to the unvaccinated cohort. This prevalence aligns with the average reported in diverse meta-analyses. Furthermore, a decrease in the overall prevalence within reinfections was observed. Certain risk factors are linked to a heightened likelihood of developing Long COVID following a COVID-19 infection such as female gender and BMI, while other factors indicate a lower risk. Notably, a protective association was identified with blood types O− and AB− and two vaccine schemes of any vaccine.

This intricate and nonspecific syndrome presents numerous aspects that remain to be fully comprehended. To establish a comprehensive understanding, standardized multi-centric trials should be undertaken, aimed at discerning the true prevalence, severity, and associations inherent to this syndrome. This effort is essential to equip medical practitioners with the insights required to offer optimal treatment strategies to patients grappling with Long COVID.

## Methodology

We executed an observational cross-sectional study within a hospital situated in Northeastern Mexico called Hospital Clinica Nova, this clinic attends a population of 50,000 steel workers and their families offering from vaccination and preventive healthcare to hospitalization and management of severe diseases. This study focused on patients with a prior occurrence of acute COVID-19 infection. The methodology adhered to the STROBE guidelines^27^. Approval for the study was obtained from the local Institutional Review Board (reference 170102022-CNa-MIa-CI), and all procedures were conducted in accordance with the ethical principles outlined in the World Medical Association's Code of Ethics (Declaration of Helsinki) pertaining to human experimentation.

The eligibility criteria encompassed adults aged 18 or older who were associated with the medical services at our hospital. To be included, participants needed to have a documented history of a COVID-19 infection confirmed through a positive diagnostic test. Acceptable tests included reverse transcriptase polymerase chain reaction (RT-PCR) or SARS-COV2 antigen test. Conversely, individuals were excluded if they exhibited suspected COVID-19 symptoms but lacked a positive diagnostic test. Also excluded were those who had experienced an acute infection within the last 4 weeks and failed to provide the comprehensive information requested during the interview.

Data collection occurred within the timeframe spanning April to May 2023. Following the signing of the informed consent, every participant underwent a comprehensive survey encompassing inquiries related to demographics (age, gender), anthropometric information [height (cm), weight (kg), body Mass Index], blood type, and patient comorbidities (Type 1 or 2 diabetes, hypertension, obstructive pulmonary disease, dyslipidemia, active cancer, previous cancer, heart failure, coronary disease, atrial fibrillation, cerebral vascular event, end stage renal disease, autoimmune disease, rheumatology disease, use of immunosuppressive medicine, organ transplantation, allergies, any medications, etc.). The number of reinfections was documented, and it was defined as a SARS-COV2 positive test, followed by a negative test that indicated patient recovery after 8–12 days of initial symptoms, and then a new infection confirmed by a positive test, followed by a negative test that indicated recovery^3^. We documented the number and type of vaccines vs. SARS-COV 2. It is important to notice that vaccination and booster in Mexico were applied by age group and not by individualized risk factors. A detailed roster of symptoms (for example: fatigue, muscle pain, alopecia, concentration problems, cough, dyspnea, loss of taste, loss of smell, thoracic pain, abdominal pain, fever, arthritis, constipation, diarrhea, loss of weight, coagulation problems, numbness, skin problems, heart failure, heart attack, etc.) experienced during the COVID-19 infection period, including their duration, was documented. If the duration of a symptom lasted more than 4 weeks in any of the infections, it was considered that the subject had Long COVID. Additionally, participants provided information about the severity of their acute symptoms and clarified if they were treated ambulatorily or if they required inpatient hospitalization or admission to an ICU unit. Every patient had an individualized interview by a trained physician. The data was anonymized and uploaded to an electronic database for analysis.

### Statistical methods

The researchers reviewed the quality control and the anonymization of the database. Normality assumption was evaluated with the Shapiro–Wilk test. Descriptive statistics such as mean, standard deviation, median, interquartile range, frequencies, and percentages were computed. We used the Chi-square test to compare the medical history of the patients and their relationship with developing Long COVID syndrome. Also, a Fisher’s exact test with a Monte Carlo approximation was used to see if there is any relation between the Long COVID syndrome presence and its symptoms with the patient’s vaccination status. A multivariable logistic regression model was used to assess the relationship between Long COVID and characteristics, including age, sex, history of allergies, BMI, blood type, number of reinfections and number of SARS-COV2 vaccines. A sample size of 750 patients was calculated, according to the primary aim, by using a logistic regression model through the G Power 3.1 The statistical analysis was performed con SPSS vs. 25. A p value < 0.05 was considered statistically significant. Missing values that occurred completely at random were handled using a complete case analysis approach.

### Ethical approval

Ethics committee/local Institutional Review Board from Hospital Clinica Nova gave ethical approval. Reference 170102022-CNa-MIa-CI.

## Data Availability

Data are available upon reasonable request to the authors.
